# Exosomal miRNAs Differentiate Chronic Total Occlusion from Acute Myocardial Infarction

**DOI:** 10.3390/ijms251810223

**Published:** 2024-09-23

**Authors:** Ji-Hye Son, Jeong Kyu Park, Ji-Hong Bang, Dongeon Kim, Inki Moon, Min Gyu Kong, Hyun-Woo Park, Hyung-Oh Choi, Hye-Sun Seo, Yoon Haeng Cho, Hun Soo Chang, Jon Suh

**Affiliations:** 1 Department of Interdisciplinary Program in Biomedical Science Major, Graduate School, Soonchunhyang University, Asan 31538, Republic of Korea; son310321@naver.com (J.-H.S.); jihong325@naver.com (J.-H.B.); 2 Department of Microbiology, College of Medicine, Soonchunhyang University, Cheonan 33151, Republic of Korea; 3 Divisions of Cardiology, Department of Internal Medicine, Soonchunhyang University Bucheon Hospital, Soonchunhyang University College of Medicine, Bucheon 14584, Republic of Korea; 135754@schmc.ac.kr (J.K.P.); byory1442@naver.com (D.K.); rokstone4330@gmail.com (I.M.); mingyu.kong@schmc.ac.kr (M.G.K.); hwpark@schmc.ac.kr (H.-W.P.); mrgud@hanmail.net (H.-O.C.); haesun@schmc.ac.kr (H.-S.S.); yhcho@schmc.ac.kr (Y.H.C.)

**Keywords:** chronic total occlusion, acute myocardial infarction, exosome, microRNA, biomarker

## Abstract

Although coronary artery occlusion can have a negative effect on the myocardium, chronic total occlusion (CTO) exhibits different clinical features from those of acute myocardial infarction (AMI). In this study, we identify the differential associations of exosomal miRNAs with CTO and AMI. Exosomes were isolated from the plasma obtained from coronary arteries of patients undergoing percutaneous coronary intervention to treat CTO (n = 29) and AMI (n = 24), followed by small RNA sequencing, target gene predictions, and functional enrichment analyses. Promising miRNA markers were validated using real-time PCR in 35 CTO, 35 AMI, and 10 normal subjects. A total of 205 miRNAs were detected in all subjects, and 20 and 12 miRNAs were upregulated and downregulated in CTO compared to AMI patients, respectively (|fold change| > 4, FDR q < 0.05). The target genes of miRNAs that were higher in CTO patients were associated with “regulation of cell cycle phase transition”, “cell growth”, and “apoptosis”. The target genes of miRNAs that were lower in CTO patients were enriched in terms such as “muscle cell differentiation”, “response to oxygen levels”, and “artery morphogenesis”. On qRT-PCR analysis, the expression levels of miR-9-5p and miR-127-3p were significantly different between CTO and AMI patients. The miRNA expression levels accurately distinguished CTO from AMI patients with 79% specificity and 97% sensitivity. The miRNA contents of plasma exosomes were significantly different between CTO and AMI patients. The miRNAs may play important roles in CTO and AMI.

## 1. Introduction

Cardiovascular diseases remain a leading cause of mortality worldwide; both chronic total occlusion (CTO) and acute myocardial infarction (AMI) are significant in this context [1]. These conditions, both of which are characterized by obstruction of coronary arteries, differ markedly in their etiologies and clinical manifestations. The pathophysiology of CTO facilitates some myocardial adaptation due to the gradual reduction in blood flow. This adaptation may limit the extent of myocardial damage compared to that associated with AMI, the rapid onset of which and the absence of pre-formed collateral circulation often trigger extensive myocardial injury, elevating the risks of severe cardiac complications such as heart failure or arrhythmia [2]. Despite the relatively better short-term clinical outcomes of CTO compared to AMI patients, those with CTO exhibit higher long-term mortality compared to patients with non-CTO coronary artery disease. This may be attributable to a greater prevalence of comorbidities, an increased incidence of risk factors, the presence of multivessel coronary artery disease, or potential biases arising when CTO lesions are unsuccessfully revascularized or revascularization is not attempted [3].

Exosomes have attracted increasing attention because they contain various molecular mediators such as proteins, phospholipids, and non-coding RNAs, including microRNAs (miRNAs) that play roles as messengers during intercellular communication [4]. It is known that exosomes mediate cardiovascular calcification [5], cardiovascular mineral metabolism [6], vascular inflammation [7], angiogenesis [8], and cardiac aging [9]. Despite the considerable evidence that exosomes play roles in cardiovascular diseases and the diagnostic and therapeutic potentials of exosomes associated with various cardiovascular diseases [10], few studies have explored the roles and associations of exosomes in/with CTO.

In recent years, miRNAs, which are small, non-coding RNA molecules, have become increasingly recognized to play crucial roles in the post-transcriptional regulation of genes [11]. miRNAs have gained attention as promising biomarkers of a range of diseases and are valuable in terms of both diagnosis and prognosis. Detection of miRNAs in plasma combines the advantages of a minimally invasive sampling method with the stability and specificity of miRNA profiles [12]. Notably, variations in miRNA expression have been observed in patients with different cardiovascular diseases. The correlations between specific miRNA signatures and cardiovascular conditions offer a more nuanced understanding of the conditions and potentially guide therapeutic interventions [13]. The roles of miRNAs have been extensively studied in the contexts of heart failure and AMI [14]. However, the expression profile and roles of miRNAs of/in CTO patients have not been thoroughly investigated.

This study identifies certain miRNAs and validates the levels thereof in the plasma of patients with CTO. It also describes the gene pathways linked to these miRNAs. Such findings will refine existing diagnostic methods and enhance our comprehension of the pathophysiology underlying chronic coronary occlusive conditions.

## 2. Results

### 2.1. Clinical Characteristics of the Study Participants

Plasma samples were obtained from the coronary arteries of patients diagnosed with CTO and AMI, and an overview of the clinical characteristics of these individuals is shown in Table 1. The prevalences of diabetes (75.9% versus 45.8%) and dyslipidemia (69.0% versus 37.5%) were notably higher in the CTO compared to the AMI group. Similarly, a history of percutaneous coronary intervention (PCI) was more common in the CTO cohort (37.9% versus 12.5%). Multivessel coronary artery disease (58.6%) and lesional calcification (65.5%) were more frequent in the CTO group. However, over a median of 595 days (interquartile range 391–952 days) of follow-up, major adverse cardiac events (MACEs), which included death from any cause, Q-wave MI, and target vessel revascularization did not differ significantly between the two groups.

### 2.2. Differential Expression of miRNAs in Exosomes of CTO and AMI Patients

**Figure 1:**
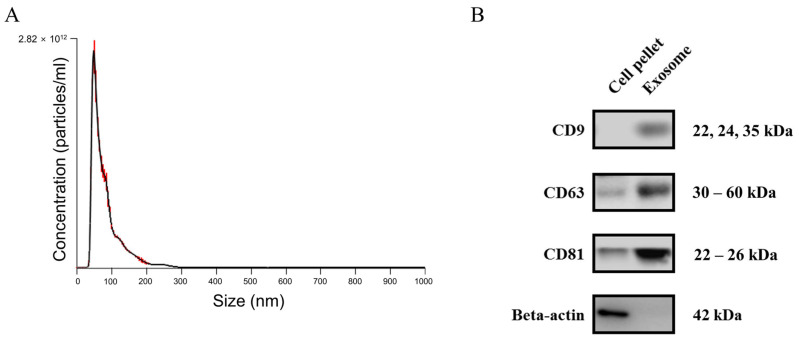
Characterization of EVs from plasma. (**A**) Diameter and concentration distribution of EVs from plasma obtained from coronary artery. (**B**) Western blot analysis of the expression of the EV-markers CD9, CD63, and CD81, and a cytoplasmic marker beta-actin in EVs from plasma.

The average diameter of exosomes isolated from plasma was 86.0 ± 0.7 nm and the average peak diameter was 54.0 ± 1.5 nm. The concentrations of released exosomes ranged from 1.07 × 10^12^ to 6.10 × 10^13^ particles/mL. Western blotting showed that the levels of the exosome markers CD9, CD63, and CD81 were higher in isolated exosomes than in crude plasma precipitates. By contrast, the cell-specific marker β-actin was not detected in isolated exosomes (Figure 1).

**Figure 2:**
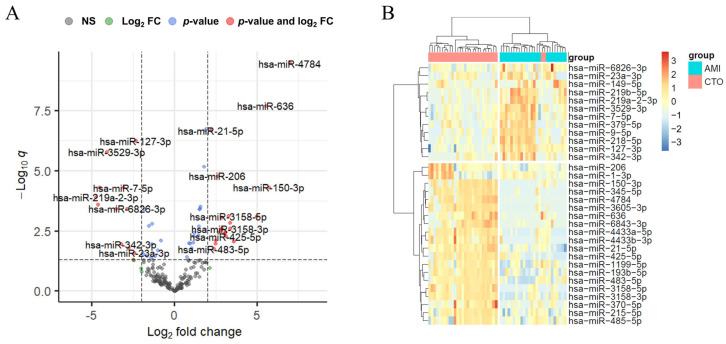
Differentially expressed miRNAs between patients with CTO and AMI. (**A**) Volcano plot shows the number and distribution of miRNAs. (**B**) Heatmap of the differentially expressed miRNAs.

To identify specific exosomal miRNAs associated with CTO as distinct from AMI, differential expression genes (DEGs) were analyzed. A total of 205 miRNAs were detected in coronary artery plasma-derived exosomes from all subjects. Among these, 20 miRNAs exhibited upregulation (fold change > 4, FDR q < 0.05) and 12 miRNAs exhibited downregulation (fold change < 0.25, FDR q < 0.05) in the exosomes of the CTO group compared to the AMI group (Figure 2 and Supplementary Table S1).

After the identification of such miRNAs, a subset of abundantly expressed miRNAs, with counts per million (cpm) exceeding 1000, was selected for further analysis. This revealed that 10 miRNAs, including miR-21-5p, miR-206, miR-215-5p, and miR-1-3p, were upregulated, whereas 3 miRNAs, including miR-9-5p, miR-3529-3p, and miR-127-3p, were downregulated in CTO compared to AMI samples (Table 2).

### 2.3. Enrichment Analyses of Biological Processes and Pathways, and the Interaction Networks of Genes Targeted by Upregulated Exosomal miRNAs in CTO Patients

**Figure 3:**
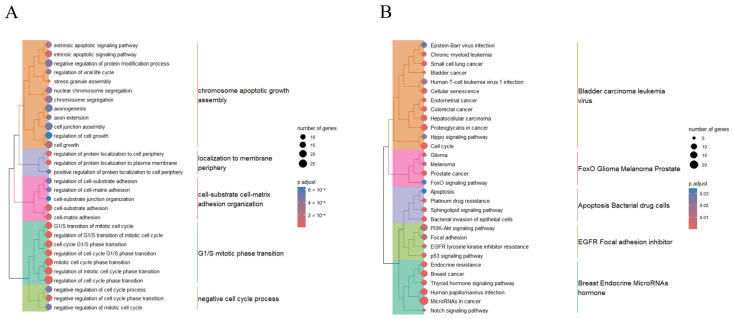
Biological process of gene ontology (**A**) and KEGG pathway (**B**) enrichment of 247 genes targeted by 10 upregulated miRNAs in patients with CTO compared to AMI patients. The color intensity indicates Maximal Clique Centrality (MCC) rank.

Of the 10 exosomal miRNAs upregulated in CTO patients, 247 target genes were identified based on the interrogation of at least two of three functionally validated miRNA target databases (miRecords, miRTarBase, and TarBase). These genes were targeted by three or more of the 10 miRNAs (Supplementary Table S2). Subsequent gene ontology enrichment analysis revealed that these 247 genes were predominantly associated with biological processes such as “regulation of cell cycle phase transition”, “cell growth”, and “intrinsic and extrinsic apoptotic signaling pathways” (Figure 3A and Supplementary Table S3). Further analysis revealed distinct clusters among these terms. Cluster 1 included terms such as “chromosome”, “apoptotic”, “growth”, and “assembly”; Cluster 2 featured terms such as “localization to”, “membrane”, and “periphery”; Cluster 3 encompassed the terms “cell-substrate”, “cell-matrix”, “adhesion”, and “organization”; Cluster 4 contained terms such as “G1/S”, “mitotic”, “phase”, and “transition”; Cluster 5 featured the terms “negative”, “cell”, “cycle”, and “process” (Figure 3A).

On KEGG pathway-enrichment analysis, the 247 target genes were enriched in the pathways of “cell cycle”, “focal adhesion”, “PI3K-Akt-signaling pathway”, and “apoptosis” (Figure 3B and Supplementary Table S4). These pathways were further clustered using representative terms. Cluster 1 included the terms “bladder”, “carcinoma”, “leukemia”, and “virus”; Cluster 2 incorporated the terms “FoxO”, “glioma”, “melanoma”, and “prostate”; Cluster 3 involved the terms “apoptosis”, “bacterial”, “drug”, and “cells”; Cluster 4 was comprised of the terms “EGFR”, “focal”, “adhesion”, and “inhibitor”; and Cluster 5 integrated the terms “breast”, “endocrine”, “MicroRNA”, and “hormone” (Figure 3B).

**Figure 4:**
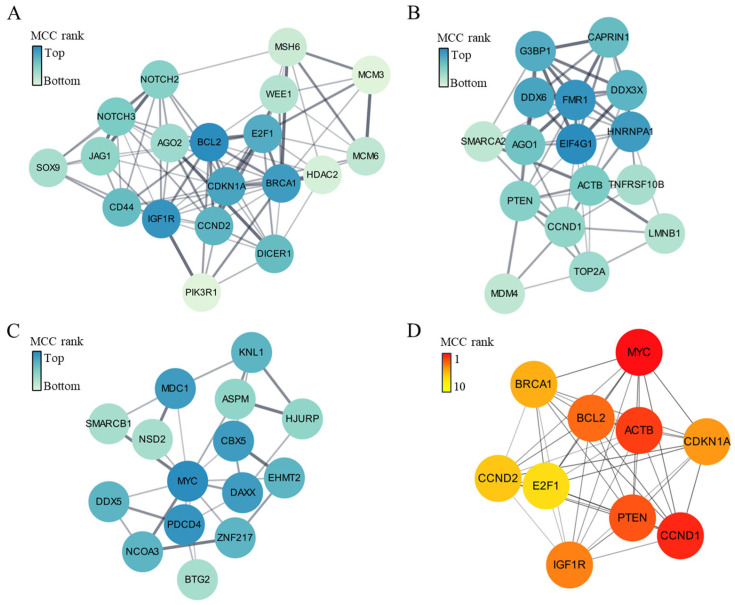
Clusters (**A**–**C**) and key genes (**D**) of protein–protein interaction network of the 247 genes targeted by 10 upregulated miRNAs in patients with CTO.

PPI network analysis of the 247 identified target genes revealed three dominant network clusters. The key genes of Cluster 1 were BCL2 (Figure 4A). The key genes of Cluster 2 were EIF4G1 and FMR1 (Figure 4B). The key genes of Cluster 3 were MYC (Figure 4C). Overall, the key genes of the entire protein–protein interaction network of the 247 genes were MYC and CCND1 (Figure 4D).

### 2.4. Enrichments of Biological Processes and Pathways, and the Interaction Network of Genes Targeted by Downregulated Exosomal miRNAs in CTO

**Figure 5:**
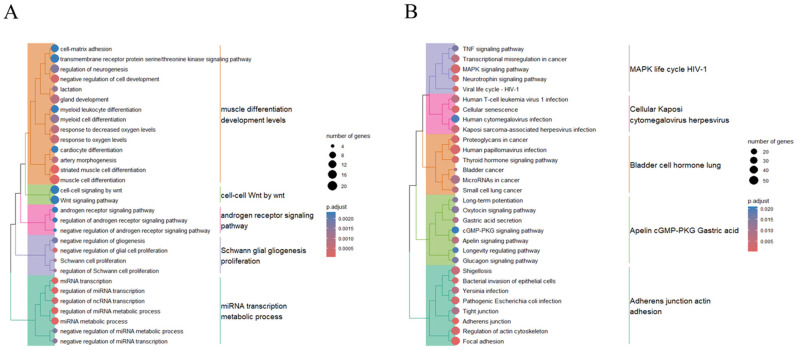
Biological process of gene ontology (**A**) and KEGG pathway (**B**) enrichment of 219 genes targeted by three downregulated miRNAs in patients with CTO compared to AMI patients.

Of the three downregulated exosomal miRNAs in CTO patients, 219 target genes were identified in at least two of the three miRNA target databases (Supplementary Table S5). Gene ontology-enrichment analysis revealed that these genes were predominantly associated with biological processes such as “muscle cell differentiation”, “response to oxygen levels”, and “artery morphogenesis” (Figure 5A and Supplementary Table S6). These terms were then grouped into clusters with representative terms, including Cluster 1 with “muscle”, “differentiation”, “development”, and “levels”; Cluster 2 with “cell–cell” and “Wnt”; Cluster 3 with “androgen”, “receptor”, “signaling”, and “pathway”; Cluster 4 with “Schwann”, “glial”, “gliogenesis”, and “proliferation”; and Cluster 5 with “miRNA”, “transcription”, “metabolic”, and “process” (Figure 5A).

On pathway-enrichment analysis, the 219 target genes were enriched in pathways such as the “MAPK-signaling pathway”, “focal adhesion”, and “tight junction” (Figure 5B and Supplementary Table S7). These pathways were further clustered by representative terms, including Cluster 1 with “MAPK”, “life”, “cycle”, and “HIV-1”; Cluster 2 with “cellular”, “kaposi”, “cytomegalovirus”, and “herpesvirus”; Cluster 3 with “bladder”, “cell”, “hormone”, and “lung”; Cluster 4 with “Apelin”, “cGMP-PKG”, “gastric”, and “acid”; and Cluster 5 with “adherens”, “junction”, “actin”, and “adhesion” (Figure 5B).

**Figure 6:**
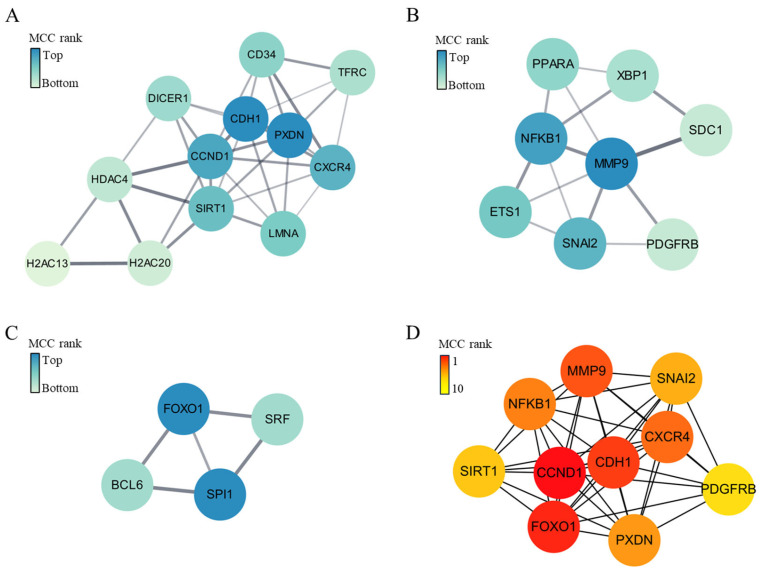
Clusters (**A**–**C**) and key genes (**D**) of protein–protein interaction network of the 219 genes targeted by 3 downregulated miRNAs in patients with CTO. The color intensity indicates Maximal Clique Centrality (MCC) rank.

PPI network analysis of the 219 identified target genes revealed three dominant network clusters. In Cluster 1, the key genes were CDH1, and PXDN (Figure 6A). The Cluster 2 key genes included MMP9 (Figure 6B). The Cluster 3 key genes were FOXO1 and SPI1 (Figure 6C). The key genes of the entire protein–protein interaction network, thus encompassing all 219 genes, were CCND1 and FOXO1 (Figure 6D).

### 2.5. Validation of the miRNA Expression Levels in Normal Control, AMI, and CTO Samples via qRT-PCR

**Figure 7:**
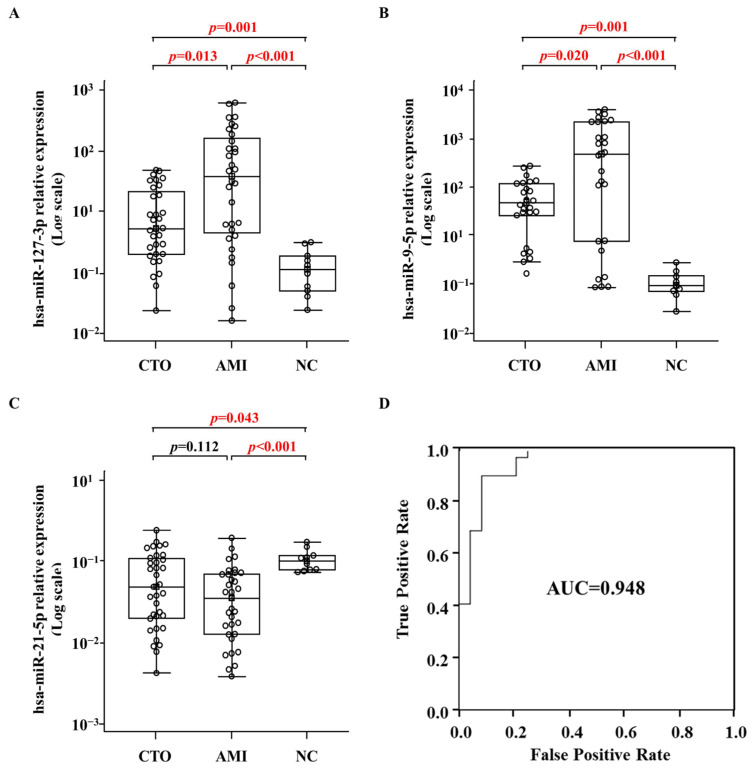
The expression levels of miR-127-3p (**A**), miR-9-5p (**B**), and miR-21-5p (**C**) in plasma EVs analyzed using qRT-PCR and ROC curve (**D**) for miR-127-3p and miR-9-5p in discrimination between the CTO and AMI (AUC = 0.948 with 79% specificity and 97% sensitivity). Box plots represent median and quartile ranges.

To validate the differentially expressed miRNAs of CTO and AMI, we selected the three miRNAs miR-21-5p, miR-9-5p, and miR-127-3p, which were related to major pathway terms of gene ontology and were of high abundance in plasma obtained from the coronary artery. We validated the expression thereof in additional patients with CTO (n = 20) and AMI (n = 20), normal controls (n = 10), and 15 patients with each of CTO and AMI randomly selected from the patients subjected to the NGS analysis (Table 1). The expression levels of miR-9-5p and miR-127-3p in plasma exosomes were significantly lower in CTO compared to AMI patients (*p* < 0.001) but significantly higher than those of normal subjects (*p* < 0.001, Figure 7A,B). The levels differed significantly among the groups (*p* < 0.05). The expression levels of miR-21-5p in plasma exosomes were significantly lower in CTO and AMI patients compared to normal subjects (*p* < 0.05), although the levels were comparable between CTO and AMI patients (*p* > 0.05, Figure 7C).

ROC analysis was used to analyze the two miRNAs and the integrative miRNA expression score accurately distinguished CTO from AMI (area under the curve = 0.948) with 79% specificity and 97% sensitivity (*p* < 0.001, Figure 7D).

## 3. Discussion

In this study, our objective was to assess differences in exosomal miRNAs isolated from the plasma of coronary arteries of patients with CTO and AMI and to elucidate their putative biological functions. Among the 205 detected miRNAs, 20 were upregulated and 12 were downregulated. The 247 target genes corresponding to the most abundant upregulated miRNAs were found to be principally associated with apoptosis and cell growth. Conversely, the target genes of downregulated miRNAs appeared to be associated with muscle cell differentiation, the response to hypoxia, and artery morphogenesis, potentially mediated by the MAPK-signaling pathways. To the best of our knowledge, this study is the first report on the associations and functional roles of exosomal miRNAs in plasma obtained from coronary arteries by comparing CTO and AMI.

Various miRNAs of exosomes are associated with the pathophysiology of AMI and CTO. It was reported that the increased levels of miR-126 and miR-199a in microvesicles isolated from femoral artery plasma were significantly associated with a lower major adverse cardiovascular event rate [15]. In the CTO context, Hakimnzadeh et al. reported that elevated levels of miR-423-5p, miR-10b, miR-30d, and miR-126 in aortic plasma of patients with inadequate collateral network development were apparent on multiplex qPCR, of which miR-30d and miR-126 exhibited a higher expression in CTO patients than healthy controls [16]. Although multiplex RT-PCR was used to detect 750 miRNAs, their observations agree with our results in that the levels of miR-423-5p and miR-30d-5p were higher in patients with CTO than AMI (Supplementary Table S1). Although the detected miRNAs differed between the Hakimnzadeh study and our work, possibly because the groups were of different sizes and the assays varied, these observations suggest that miRNAs play roles in the development and pathophysiology of CTO.

Our gene ontology and pathway-enrichment analysis showed that the genes targeted by miRNAs that were upregulated in CTO compared to AMI patients may be involved in cell survival, proliferation, differentiation, and apoptosis. In an ischemic environment, it is known that cardiac myocytes are lost because of cell death and apoptosis [17], and that cell proliferation and survival then increase to maintain homeostasis [18]. In addition, the differentiation of precursor cells into cardiac myocytes, endothelial cells, and fibroblasts is then induced to restore cardiovascular structure and function [19], which may be mediated by BCL2 and Akt signaling [20]. The well-known PI3K-Akt-signaling pathway is considered cardioprotective, promoting cell survival and inhibiting apoptosis [21]. In the context of AMI, the PI3K-Akt pathway may also be activated as part of the cellular response that protects cardiac myocytes from ischemic injury [22]. In contrast, angiotensin alleviates cardiac remodeling by inhibiting the PI3K-Akt pathway [23]. Our results show that this pathway was likely to be downregulated in CTO compared to AMI patients, which suggests that the molecular pathways that protect against ischemic damage may be less active in CTO than in AMI patients. Furthermore, as shown by the network analysis (Figure 4), BCL2 was the main target of exosomal miRNAs in the plasma of CTO patients. Thus, our observations agree with previous reports on the role of apoptosis and the PI3K-Akt pathway in AMI.

By contrast, our results showed that the downregulated exosomal miRNAs in CTO (compared to AMI) targeted principally genes involved in muscle cell differentiation, artery morphogenesis, the response to oxygen levels mediated by the MAPK-signaling pathway, focal adhesion, and cell junctions. The endothelial cell junctions, including adherens and tight junctions, play roles in the control of vascular integrity and function, as well as angiogenesis and vascular remodeling [24]. Further, the MAPK-signaling pathway was reported to be involved in the angiogenesis triggered by ischemic conditions [25]. Collectively, our results suggest that the molecular mechanisms of angiogenesis, artery morphogenesis, and vascular function may be activated in CTO compared to AMI patients, although direct evidence for the roles played by MAPK signaling in CTO has not yet been derived. Recently, Gao et al. revealed that the lncRNA-miRNA-mRNA network of CTO might be related to angiogenesis mediated by the MAPK-signaling pathway, the HIF-1-signaling pathway, EGFR tyrosine kinase inhibitor resistance, and embryonic organ development, similar to our observations [26].

To validate the different miRNA levels, we selected three miRNAs, miR-21-5p, miR-9-5p, and miR-127-3p, by reference to their read counts and the ontology terms of their target genes. Of these, the levels of miR-127-3p and miR-9-5p were higher in CTO and AMI patients than in normal subjects and lower in CTO than in AMI patients (Figure 7). miR-127-3p has been reported to inhibit the proliferation of cancer cells [27] and myocytes [28], and to regulate autophagy in the hypoxic–ischemic cortex [29]. miR-9-5p is associated with the proliferation of various cancer cells [30], organ fibrosis [31], and the neuroinflammation of hypoxic–ischemic brain injury [32]. Our qPCR results showed that the level of miR-21-5p expression was lower in both CTO and AMI patients compared to normal subjects and comparable between CTO and AMI patients. Recently, a decreased expression of miR-21-5p has been associated with endothelial and smooth muscle progenitor cell differentiation from amniotic fluid stem cells [33]; miR-21-5p upregulation restored the cardiomyocyte proliferation that was inhibited by high glucose [34]. Taken together, our results suggest that these miRNAs may play important roles during AMI or CTO development under ischemic conditions possibly by regulating cardiomyocyte differentiation, proliferation, and survival.

This study had several limitations. First, we focused on the abundant miRNAs in exosomes and target genes shared by multiple miRNAs, which limited our insight into the associations between exosomal miRNAs and CTO and may underestimate the roles of miRNAs expressed at medium levels or as trace amounts. Second, we could not evaluate the biological roles played by miRNAs, such as the differential regulation of target gene expression and function in terms of CTO development and pathophysiology. Thus, a further study using transcriptome profiling will be necessary to confirm the levels of target gene expression, and a functional study is needed to reveal the roles of the target genes in CTO. Third, we did not specifically analyze the effect of sex on the differential expression of exosomal miRNAs, as the NGS- and qPCR-validated subjects did not show significant sex bias in the distribution of CTO, AMI, and normal subjects (Table 1). However, since the importance of sex in the regulation of various cardiac diseases has recently emerged [35], further evaluation of sex-based miRNA profiles using larger sample sizes could provide more accurate insights into the role of miRNAs in CTO and AMI. Finally, we focused on only miRNA in exosomes. We did not assess the proteins and other non-coding RNAs that are also contained in exosomes. Additional multi-omics profiling of the characteristics of exosomes of CTO and AMI patients would yield more precise knowledge on the roles played by exosomes in cardiovascular diseases. Despite these limitations, to the best of our knowledge, this study is the first to suggest possible associations between specific miRNAs of exosomes and the development and regulation of CTO compared to AMI. We performed large-scale NGS to derive these results.

Our results show that the miRNA patterns differed significantly between various groups, suggesting that the expression levels of miRNAs could serve as biomarkers distinguishing CTO from AMI. Furthermore, the functional enrichment analyses showed that the circulating exosomal miRNAs may regulate the functions of key modulators involved in cell proliferation and angioneogenesis in the context of CTO development and pathophysiology. These functional miRNAs and pathways could serve as potential candidates for novel therapeutics and targets to modulate the progression of CTO and AMI.

## 4. Materials and Methods

### 4.1. Study Subjects and Plasma Preparation

Plasma samples from patients who underwent percutaneous coronary intervention (PCI) to treat CTO (n = 35) and AMI (n = 35), and those with normal coronary arteries (NCs) (n = 10) were retrieved from the biobank of Soonchunhyang University Hospital, Bucheon, Korea (schbc-biobank-2021-011) after approval of the study protocol by the Soonchunhyang University Hospital Ethics Committee (approval no. SCHBC 2021-07-047). All patients underwent coronary angiography via the radial or femoral artery. CTO was identified as a coronary artery blockage with a Thrombolysis in Myocardial Infarction (TIMI) flow grade of 0 that persisted for at least 3 months as determined by the clinical history and prior angiography. In cases lacking clear evidence of duration, CTO diagnosis relied on the angiographic characteristics of the EuroCTO Club guideline [36]. AMI is characterized by myocardial ischemia that causes myocardial injury, diagnosed via elevated high-sensitivity troponin levels, typical symptoms, indicative electrocardiographic alterations, and angiographic signs of new myocardial damage or a wall motion abnormality [37]. Blood samples were obtained from patients during coronary angiography.

All 53 patients of the CTO and AMI groups underwent exosome isolation from plasma samples and miRNA expression profiling. Then, the data on each of 20 randomly selected patients of the CTO and AMI groups were validated by quantitative real-time polymerase chain reaction (qRT-PCR). During such validation, we included analyses of 15 newly identified patients with CTO or AMI and an additional 10 NC individuals. Finally, miRNA qRT-PCR analysis was performed for a cohort of 80 individuals, comprising 35 patients with CTO, 35 with AMI, and 10 NCs.

### 4.2. Isolation and Characterization of Exosomes

In line with the manufacturer’s instructions, exosomes were isolated from 1 mL samples of plasma using the miRCURY exosome isolation kit (Qiagen, Hilden, Germany) after filtration of the plasma samples through 0.22 µm-pore-sized syringe filters (Sartorius Stedim Biotech, Aubagne, France). All exosomes were stored at −80 °C immediately after isolation until further experimentation.

Nanoparticle tracking analysis (NTA) was performed to determine the concentrations and sizes of isolated exosomes using a NanoSight NS300 instrument (Malvern Panalytical Ltd., Malvern, UK) [38]. Western blotting was used to identify exosome proteins [39]. Such proteins (15 µg/well) were resolved by 10% polyacrylamide gels, transferred to nitrocellulose membranes, blocked in 5% skim milk, and probed with an anti-CD9 monoclonal antibody at 1:500 dilution (Cell Signaling Technology, Danvers, MA, USA), an anti-CD63 monoclonal antibody at 1:1000 dilution (Santa Cruz Biotechnology, Dallas, TX, USA), an anti-CD81 monoclonal antibody at 1:500 dilution (Santa Cruz Biotechnology), and an anti-β-actin monoclonal antibody at 1:10,000 dilution (Sigma–Aldrich, St. Louis, MO, USA). The membranes were then incubated with a horseradish peroxidase-conjugated secondary antibody at 1:5000 dilution (GenDEPOT, Baker, TX, USA). Target proteins were detected using an enhanced chemiluminescence method (Amersham Pharmacia Biotech, Buckinghamshire, UK) and visualized using the digital Azure Biosystems C280 system (Azure Biosystems, Dublin, CA, USA).

### 4.3. Procedures for Small RNA Sequencing

Total RNA was isolated from exosomes into the TRIzol LS reagent (Ambion, Life Technology, Carlsbad, CA, USA) according to the manufacturer’s instructions. RNA quality was assessed with the aid of an Agilent 2100 Bioanalyzer fitted with the RNA 6000 Pico Chip (Agilent Technologies, Amstelveen, the Netherlands); RNA quantification employed the Qubit assay system (Thermo Fisher Scientific, Waltham, MA, USA).

Libraries of control and test RNAs were constructed using NEBNext Multiplex Small RNA Library Prep kits (New England BioLabs, Inc., Ipswich, MA, USA) according to the manufacturer’s instructions. The yields and size distributions of small RNA libraries were assessed with the aid of the Agilent 2100 Bioanalyzer instrument running the high-sensitivity DNA assay (Agilent Technologies). High-throughput sequences were generated using the NextSeq 500 system employing single-end 75 sequencing.

Sequence reads were mapped by bowtie2 software that yielded bam files. A mature miRNA sequence served as a reference when mapping. Read counts mapped onto mature miRNA sequences were extracted from the alignment file using bedtools v2.25.0 [40] and Bioconductor [41] in the R program (R Development Core Team, 2016). The read counts were used when determining the expression level of miRNAs. The count per million (CPM) and the trimmed mean of the M-values (TMM) normalization method were used to compare samples.

### 4.4. Differentially Expressed Gene Analysis and Target Gene Analysis

Differentially expressed gene (DEG) analysis of the exosomal miRNAs of the CTO and AMI groups was performed using the generalized linear model quasi-likelihood F-test (glmQLFTest) function of the EdgeR package of R [42]. The target genes of upregulated or downregulated miRNAs in the CTO group were identified using a multiMiR package [43]. Gene ontology and KEGG pathway-enrichment analyses were performed employing the clusterProfiler [44]. The similarities between the obtained gene ontology or KEGG pathways were calculated and visualized using the enrichplot package (https://bioconductor.org/packages/enrichplot accessed on 15 February 2024).

### 4.5. miRNA qRT-PCR Analysis

During miRNA-specific reverse transcription, miRNA was reverse-transcribed to cDNA using the TaqMan^®^ MicroRNA Reverse Transcription Kit (Applied Biosystems, Waltham, MA, USA) and specific primers according to the manufacturer’s instructions. Reverse transcription was performed as follows: 30 min at 16 °C and 30 min at 42 °C, followed by 5 min at 85 °C. The resulting cDNAs were stored at −80 °C until use.

qRT-PCR was used to quantify the expression levels (in duplicate) of mature miRNAs using TaqMan^®^ MicroRNA Assay kits and the TaqMan^®^ Universal Master Mix (Applied Biosystems) with the aid of an ABI 7500 real-time PCR system according to the manufacturer’s instructions. miR-30e-5p and miR-151a-3p served as endogenous controls when normalizing the expression levels of target miRNAs. The relative quantification (Rq) of each miRNA expression level was calculated using the 2^−ΔΔCT^ threshold cycle method. qRT-PCR was performed as follows: 2 min at 50 °C and 10 min at 95 °C, followed by 40 cycles at 95 °C for 15 s and 60 °C for 1 min.

### 4.6. Statistical Analyses

For normally distributed parameters as revealed by the Shapiro–Wilk test, one-way analysis of variance (ANOVA) and the *t*-test were applied. For variables with skewed distributions, between-group comparisons were performed using the Kruskal–Wallis test followed by the Mann–Whitney U-test (post-hoc analysis). After conditional backward logistic regression analysis, integrative miRNA expression scores were computed for all retained miRNAs employing the regression equation: miRNA expression score = ∑(odds ratios × levels of miRNA expression). Subsequently, a receiver operating characteristic (ROC) analysis was conducted to evaluate the accuracy of the miRNA expression score in terms of discriminating CTO from AMI. All statistical analyses employed SPSS software (version 24.0; SPSS Inc., Chicago, IL, USA), and the significance threshold was set at a *p*-value < 0.05.

## Figures and Tables

**Table 1:** Clinical characteristics of the study subjects.

Variable	NGS			Validation (qPCR)			
	AMI	CTO	*p*-Value	CTO	AMI	NC	*p*-Value
No.	24	29	-	35	35	10	-
Sex (Male/Female)	20/4	22/7	0.504	30/5	30/5	8/2	0.894
Age (years)	63.5 (52.5–72)	64.0 (61.0–69.5)	0.714	64 (59–69)	63 (51–67)	65 (63–68)	0.692
Smoking (%)	12 (50.0%)	11 (37.9%)	0.378	15 (42.9%)	19 (54.3%)	2 (20%)	0.149
Body mass index (kg/m^2^)	24.25 ± 0.38	25.70 ± 0.55	0.054	25.72 ± 0.61	24.26 ± 0.36	26.92 ± 0.73	0.009
Hypertension (%)	15 (62.5%)	22 (75.9%)	0.292	27 (77.1%)	20 (57.1%)	9 (90%)	0.064
Diabetes (%)	11 (45.8%)	22 (75.9%)	0.025	23 (65.7%)	15 (42.9%)	3 (30%)	0.057
Dyslipidemia (%)	9 (37.5%)	20 (69.0%)	0.022	23 (65.7%)	17 (48.6%)	9 (90%)	0.046
Chronic kidney disease (%)	4 (16.7%)	6 (20.7%)	0.709	7 (20%)	4 (11.4%)	0 (0%)	0.234
Previous PCI (%)	3 (12.5%)	11 (37.9%)	0.037	13 (37.1%)	4 (11.4%)	1 (10%)	0.022
Previous MI (%)	2 (8.3%)	6 (20.7%)	0.211	8 (22.9%)	3 (8.6%)	0 (0%)	0.089
Previous CVA (%)	5 (20.8%)	7 (24.1%)	0.775	8 (22.9%)	1 (2.9%)	1 (10%)	0.039
Previous PAD (%)	1 (4.2%)	5 (17.2%)	0.135	4 (11.4%)	1 (2.9%)	0 (0%)	0.228
hs-CRP (mg/dL)	0.33 (0.09–1.15)	0.17 (0.07–0.60)	0.537	0.16 (0.07–0.48)	0.19 (0.07–0.77)	0.09 (0.06–0.1)	0.099
LDL-cholesterol (mg/dL)	108.5 (77.3–128.0)	110.0 (79.5–132.5)	0.782	94 (61–116)	114 (94–130)	103.5 (62.8–120.8)	0.070
Creatinine (mg/dL)	1.00 (0.93–1.28)	0.90 (0.85–1.10)	0.070	1(0.9–1.1)	1.1 (0.95–1.2)	-	0.482
Pre LVEF (%)	52.0 (43.3–61.5)	56.0 (43.5–60.5)	0.761	53 (46–59)	47 (44–52)	-	0.081
FU LVEF (%)	58.5 (46.0–66.8)	50.0 (46.0–56.0)	0.262	50 (46.5–56.25)	53 (47–62)	-	0.579
Target vessel (culprit lesion)							
LAD	12 (50.0%)	10 (34.5%)	0.220	12 (34.3%)	14 (40%)	-	0.446
RCA	6 (25.0%)	5 (17.2%)		6 (17.1%)	9 (25.7%)	-	
LCX	6 (25.0%)	14 (48.3%)		17 (48.6%)	12 (34.3%)	-	
Coronary artery disease (LM excepted)							
1VD	9 (37.5%)	2 (6.9%)	<0.001	6 (17.1%)	17 (48.6%)	-	0.001
2VD	12 (50%)	10 (34.5%)		12 (34.3%)	14 (40%)	-	
3VD	3 (12.5%)	17 (58.6%)		17 (48.6%)	4 (11.4%)	-	
Calcification (%)	4 (16.7%)	19 (65.5%)	<0.001				
MACE	1 (4.2%)	6 (20.7%)	0.077				
TVR (%)	0 (0%)	3 (10.3%)	0.105				
Death (%)	1 (4.2%)	1 (3.4%)	0.891				

**Table 2:** Top 10 significantly upregulated and 3 downregulated miRNA between groups.

miRNA	AMI	CTO	glmQLFTest				
			logFC	logCPM	F	*p*-Value	FDR
hsa-miR-3605-3p	6.72 ± 8.63	11.67 ± 12.11	4.97	10.84	18.01	<0.001	0.001
hsa-miR-345-5p	7.23 ± 9.42	10.8 ± 10.65	3.58	10.02	10.87	0.002	0.009
hsa-miR-3158-3p	8.14 ± 8.85	11.5 ± 11.17	3.36	10.74	14.48	<0.001	0.003
hsa-miR-1-3p	12.48 ± 12.74	15.58 ± 16.5	3.09	14.84	13.42	0.001	0.004
hsa-miR-485-5p	8.85 ± 9.35	11.7 ± 13.29	2.86	10.98	14.47	<0.001	0.003
hsa-miR-215-5p	9.83 ± 10.85	12.68 ± 13.33	2.85	11.97	17.59	<0.001	0.001
hsa-miR-206	10.63 ± 10.17	13.22 ± 13.67	2.59	12.54	31.28	<0.001	<0.001
hsa-miR-1199-5p	8.26 ± 8.93	10.74 ± 10.88	2.49	10.07	11.17	0.001	0.008
hsa-miR-483-5p	9.37 ± 10.47	11.74 ± 11.36	2.38	11.08	8.92	0.004	0.018
hsa-miR-21-5p	9.61 ± 9.13	11.82 ± 11.15	2.21	11.19	47.81	<0.001	<0.001
hsa-miR-127-3p	10.87 ± 10.42	8.53 ± 7.87	−2.35	10.04	43.57	<0.001	<0.001
hsa-miR-3529-3p	11.11 ± 11.78	7.03 ± 7.34	−4.11	10.07	39.34	<0.001	<0.001
hsa-miR-9-5p	12.19 ± 12.26	7.59 ± 8.24	−4.62	11.11	22.1	<0.001	<0.001

## Data Availability

The original data presented in the study are openly available in National Library of Medicine (BioProject accession: PRJNA1142434) at https://dataview.ncbi.nlm.nih.gov/object/PRJNA1142434?reviewer=g07scni4k7n6hqq7o384ptp1bl accessed on 1 August 2024.

## Supplementary Materials

The following supporting information can be downloaded at: https://www.mdpi.com/article/10.3390/ijms251810223/s1.
